# Editorial for the Special Issue on “Nanodevices for Microwave and Millimeter Wave Applications”

**DOI:** 10.3390/mi11050477

**Published:** 2020-05-02

**Authors:** Isabelle Huynen

**Affiliations:** Institute of Information and Communication Technologies, Electronics and Applied Mathematics (ICTEAM), Université catholique de Louvain, Place du Levant 3/P.O. Box L5.03.02, 1348 Louvain-la-Neuve, Belgium; isabelle.huynen@uclouvain.be

Initially inspired by the work of Richard Feynman in 1959 during his famous talk “There is plenty of room at the bottom”, nanoscience and nanotechnology have moved during the 2000s from laboratory developments to daily life applications. The nanoworld, as understood today, is at the frontier between the level of atoms and molecules, governed by quantum physics and the macroworld where materials have bulk properties resulting from the assembly of billions of atoms.

In this context, I am pleased as a Guest Editor to introduce this Special Issue focused on the interaction between nanoscale dimensions and centimeter to millimeter wavelengths. Such interaction is efficient for the design and fabrication of devices showing enhanced performances. This issue includes seven novel contributions in the field. The applications cover electronics, sensors, signal processing through amplification and the switching, mixing and broadcasting of signals, all exploiting nanoscale/nanotechnology at microwave and millimeter waves.

Heredia et al. [1] presented a frequency-reconfigurable 130nm SiGe:C BiCMOS, very compact Low Noise Amplifier (LNA) in the range 120–140 GHz. The LNA design is based on an Heterojunction Bipolar Transistor (HBT)-based switch instead of a RF-MEMS switch. A systematic procedure was applied to design the input-, inter-stage- and output-matching networks in order to obtain a perfectly balanced gain and noise figure at both frequency states. The measured gain and noise figures were 14.2/14.2 dB and 8.2/8.2 dB at 120/140 GHz, respectively, in very good agreement with circuit/electromagnetic co-simulations. 

In [2] Rydosz et al. proposed a microwave gas sensor based on a 250 nm-tick SnO_2_ film. Its sensitivity to acetone was improved by applying UV illumination, which emphasized the sensor’s response to lower gas concentrations. As a result, detection was obtained for a wide range of gas concentrations. Various experimental conditions were tested. The highest sensitivity was obtained for a UV (375 nm) current of 10 mA at a continuous wave.

In [3] Tripon-Canseliet et al. achieved the microwave extraction of the electrical impedance of vertically aligned multi-wall carbon nanotube (VA MWCNT) bundles/forests grown on a silicon substrate. Dedicated resonating devices were designed for antenna application, operating around 10 GHz and benefiting from a natural inductive/capacitive behavior or complex conductivity in the microwave domain. As obtained from the S-parameter measurements, the capacitive and inductive behaviors of VA MWCNT bundles were deduced from the device frequency resonance shift.

Van Kerckhoven et al. [4] developed and compared two laser-assisted processes for the fabrication of microwave devices based on metallic nanowire arrays loaded inside porous alumina templates, allowing the realization of substrate integrated waveguide (SIW)-based devices. A nanowired SIW was firstly presented. It operated between 8.5 and 17 GHz, corresponding to the first and second cut-off frequencies of the waveguide, respectively. Then, a nanowired SIW isolator was demonstrated. It showed a nonreciprocal isolation of 12 dB, observed in absence of a DC magnetic field, which was achieved through an adequate positioning of ferromagnetic nanowires inside the waveguide.

In the millimeter waves range, a novel J band (i.e., between 220 and 325 GHz) MEMS switch was developed by Zhang et al. [5]. In order to improve the isolation in the “DOWN” state, the capacitance value in the “DOWN” state was increased by a thin isolation layer Si_3_N_4_ on the bottom plate of the switch with a thickness of ~100 nm. The switch was actuated under a voltage of ~30 V. More importantly, the switch achieved a low insertion loss of ~1.2 dB at 220 GHz and <~4 dB from 220 GHz to 270 GHz in the “UP” state, and an isolation of ~16 dB from 220 GHz to 320 GHz in the “DOWN” state. 

In the field of microwave antennas Baba et al. [6] proposed a wideband planar and integrated a feeding structure for resonant cavity antennas (RCAs) including partially reflective structures, as a convenient alternative in applications requiring high directivity and bandwidth. A maximum antenna gain of 16.7 dBi was obtained with a directivity bandwidth covering nearly the entire Ku-band.

In the field of signal processing Wu et al. [7] developed a 135–190 GHz self-biased broadband frequency doubler based on planar Schottky diodes having an epitaxial layer thickness of 260 nm. The measured results showed that the doubler exhibited a 3 dB bandwidth of 34% from 135 GHz to 190 GHz, with a conversion efficiency of above 4% when supplied with 100 mW of input power. A 17.8 mW peak output power with a 10.2% efficiency was measured at 166 GHz when the input power was 174 mW, in agreement with the simulations. 

I would like to take this opportunity to thank all the authors for submitting their papers to this Special Issue. I also want to thank all the reviewers for dedicating their time and helping to improve the quality of this Special Issue.
